# Humane Methods of Wedging

**Published:** 1909-08-15

**Authors:** S. E. Davenport


### HUMANE METHODS OF WEDGING.

BY S. E. DAVENPORT, D.D.S., M.D.S.

The wedging of teeth, like other departments of operative dentistry, has been subject to fads and fancies, but the many changes of method have gradually resulted in considerable improvement.

At the present time there are in general use for wedging a number of materials, all of which are humane in their action, when properly used, but differing in result according to the shape of the teeth being separated.

Long experience and considerable study of the subject enables the writer to state very positively that teeth can be separated a considerable distance with practically no pain and but little soreness if the proper wedging material be chosen and the gum not encroached upon.

The last two clauses of the last sentence really mean the same thing, for if the dentist has studied the question and understands the need of each situation as presented, he will be able to select a wedging material which will do the work without coming in contact with the soft parts.

Orthodontists and dentists pretty generally agree that the teeth can be moved long distances without special discomfort to the patient if their wires and ligatures are kept away from the gum.

While the teeth which dentists are called upon to wedge present conditions of considerable variety, they may for present purposes be divided into two classes. First, teeth whose approximate surfaces are nearly parallel, such teeth being large at the gum margin; second, those teeth more ideal in shape, with constricted necks and approximal surfaces tapering sharply to knuckles firmly in contact near the masticating surface.

Class one, with sides nearly parallel, can probably be better wedged by the use of tape, made of either cotton or linen, so waxed or permeated by paraffine or other substance as to prevent much absorption of the food particles which makes unwaxed tape offensive in a short time.

Nearly thirty years ago Dr. L. D. Shepard, of Boston, presented this method of wedging in a very scientific manner before the Connecticut Valley Dental Society at Springfield, Mass.

The writer believes Dr. Shepard to have been the first to introduce to the profession a tape which could be used without becoming offensive in the mouth and a system so complete and accurate that either dentist or patient, when instructed, could obtain gradual results with a minimum of discomfort.

Dr. E. A. Bogue, of New York City, has perfected this method of wedging to a high degree and discovered in Paris years ago, a thin tape with glazed surface and fine texture made of linen and cotton mixed, which has proved most satisfactory.

Among others who have given considerable attention to this important matter may be mentioned, Dr. C. Frank Bliven, of Worcester and Boston, who has also, the writer believes, devised a special tape for the purpose.

Added emphasis may worthily be given to the fact that patients who are at all skillful with their fingers may be instructed regarding this tape method of wedging and enabled to prepare spaces for filling, thereby saving the time of all concerned.

The detail of this instruction is probably unnecessary, as all, no doubt, are familiar with the plan of adding one or two thickness of tape when the wedge is changed either by the patient or the dentist's assistant. The use of tape, however, is not indicated with teeth of class two as described above, where the approximal surfaces touch at a single point only, near the masticating surface, for with such teeth, tape is either placed with difficulty, or, if well placed, is likely soon to be forced into the gum.

The truth of this has been recognized by many operators who have obviated the difficulty in different ways.

Dr. Carl C. Smith, of New York, uses for wedging teeth of class two, linen carpet thread (Barbour's Irish Flax) of various sizes, which he ties either single or double, according to the need of the individual case, around the knuckles between the teeth. This material does not swell when wet, which means that its wedging action depends upon the force with which it is tied.

Dr. H. Griffin Marshall, of New York, has for years used with success the following method:

He passes a silk thread between the teeth and before tieing the silk, packs in a few fibres of absorbent cotton. By knotting the silk around the cotton, the wedge is prevented from slipping into the gum and the expansion of the cotton is effective in separating the teeth.

Men all over the world have for years recognized the difficulty of wedging teeth in class two and have experimented with many materials in the effort to so secure the wedge that the very success of its action would not cause it to work into the gum.

Most of these experiments have been with materials which could be tied between the teeth, either with or without a few fibres of cotton, the knuckles of the teeth being depended upon to prevent a movement of the wedge toward the gum. The use of cotton is objectionable as it becomes offensive in a few hours and most of the other materials depend for their action upon the force with which they are tied, rather than upon any special swelling property.

The best way to test all these materials for this special property, outside the mouth, is to place a carefully measured length of the material, say, one inch, in water for twenty-four or forty-eight hours. If it has shrunk to even seven-eighths of its original length in twenty-four hours it will have increased its calibre correspondingly, and for both reasons, therefore, is a valuable wedging material for class two.

"Dr. Isaac B. Davenport, of Paris, France, noticed in the mouth of a patient who had just arrived in one of the steamers from Boston, a wedge consisting of a double silk, tied with fibres of cotton between. It instantly occurred to Dr Davenport that fish-line which he was then using for regulating purposes, would be very valuable also for wedging, and after experimenting with different sizes, he came to the conclusion that it was better to use the line without cotton. The method is especially applicable to those cases where the teeth are snugly in contact at the masticating surface with a V-shaped space at the gum, and where it is almost impossible to apply tape, wood or rubber wedges without having them slip down against the gum. * * * While sufficient space for filling may often be gained in twelve hours with but little soreness, one line will continue swelling for two or three days, where extensive wedging is needed. The method has been of the greatest service in my hands for many reasons, the most important of which is that the gum is not encroached upon, inflamation of the gum being, in my opinion, the principal cause of soreness in wedging. My experience in regulating teaches me that teeth can be moved for a considerable distance without much soreness, provided the gum is not injured. * * * One great advantage of this material over tape or absorbent cotton used in wedging is that it does not become offensive when left in the mouth for many days."

It will be noticed that the use of the fish-line method is indicated in the class of teeth which has heretofore been most difficult to wedge by any known method, which makes the fish-line or like material of the greatest value.

In other words, teeth which are so tightly knuckled that it is almost impossible to get any material between them are easily wedged by the fish-line method and without danger of the material getting into the gum.

The objections to this method are the expense of the fish-line and the difficulty of procuring it, and as long as these objections remain in force the use of the method cannot become at all general.

The fish-line may be had of Abbey & Imbrie, 18 Vesey Street, New York City.

Even if there were a number of places where good fish-line could be purchased in this country, it is imported and its cost would be quite an item in a country practice where low fees govern.

For some time the writer, recognizing the objections referred to, as well as the great value of the method, has experimented with many materials, hoping to get something as effective as fish-line, which would be inexpensive and which could be obtained anywhere, even in an ordinary country store.

He takes great pleasure in presenting as the material best adapted for the purpose, which he has been able to find up to the present time, Clark's O. N. T. crochet cotton No. 15. This well known material can be purchased at the New York City department stores for five cents a spool of two hundred yards.

It does not continue to shrink and swell in moisture for three days as does fish-line, but repeated tests prove that one inch of the material becomes seven-eights of an inch when immersed in water for twenty-four hours and there is a slight shrinkage during the second twenty-four hours.

The successful action of this material depends even more than does the fish-line upon the way it is tied.

In almost every case it should be used double in accordance with the following directions:

A piece of waxed dental floss about six inches long is doubled in the center and through the loop made is threaded a piece of the wedging material, also about six inches long.

One strand of the dental floss is passed between the teeth to be wedged and then the other, the looped end containing the wedging thread being placed on the side of the teeth most convenient. The floss silk is then pulled between the teeth until the loop of the wedging material appears, when the silk is pulled out of the loop and thrown aside.

One free end of the wedge material is then brought over above the teeth and threaded through the loop, after which an end is taken in each hand and pulled tightly.

Care should be taken to draw the loop until it comes between the teeth and the force used draws the material away from the gum and around the knuckles of the two teeth. A square knot is then tied on the cheek side and the ends cut, not too closely to the knot.

It is important that the square knot should be used so that the material will not untie, or the knot slip. If much space is required the wedge may be changed two or three times and sometimes teeth are so placed that the best results are gained by using the line wedge first, to gain a little space and then following with tape.

Dentists of the old school, though very skilful, were not particularly careful of their patients, and their methods of wedging, while positive, could not be called humane. In these days more attention is being paid to the comfort of patients and one wayin which dentists may make themselves more useful and more popular is by the adoption of humane methods of wedging.—*The Journal.*

